# Management of Intrauterine Device-Associated Actinomycosis

**DOI:** 10.1155/S1064744993000298

**Published:** 1993

**Authors:** Ashwin Chatwani, Soheil Amin-Hanjani

**Affiliations:** 1 Department of Obstetrics Gynecology, and Reproductive Sciences Temple University School of Medicine Philadelphia PA USA; 2 Temple University Hospital Department of OB/GYN & RS 3401 North Broad Street, 7-OPD Philadelphia PA 19140 USA

## Abstract

*Objective:* To assess various methods of management of actinomyces-like organisms associated with intrauterine devices.

*Methods:* A retrospective chart review of 173 patients with intrauterine device-associated actinomyces- like organisms detected on Pap smear was performed. The patients were managed by IUD removal with or without antibiotic therapy, antibiotic therapy alone, or no treatment at all.

*Results:* The success rate as reflected in negative follow-up smear was 100% for IUD removal combined with antibiotics, 97.4% for IUD removal alone, and 36.8% for antibiotics therapy alone.

*Conclusions:* The best way to manage intrauterine device-associated actinomyces-like organisms is removal of the device with or without antibiotics.


Infectious Diseases in Obstetrics and Gynecology 1:130–133 (1993)
© 1993 Wiley-Liss, Inc.

### Management of Intrauterine Device-Associated Actinomycosis

Ashwin Chatwani and Soheil Amin-Hanjani

*Department of Obstetrics, Gynecology, and Reproductive Sciences, Temple University School of Medicine, Philadelphia, PA*

ABSTRACT

**Objective:** To assess various methods of management of actinomyces-like organisms associated with intrauterine devices.

**Methods:** A retrospective chart review of 173 patients with intrauterine device-associated actinomyces-like organisms detected on Pap smear was performed. The patients were managed by IUD removal with or without antibiotic therapy, antibiotic therapy alone, or no treatment at all.

**Results:** The success rate as reflected in negative follow-up smear was 100% for IUD removal combined with antibiotics, 97.4% for IUD removal alone, and 36.8% for antibiotics therapy alone.

**Conclusions:** The best way to manage intrauterine device-associated actinomyces-like organisms is removal of the device with or without antibiotics. © 1993 Wiley-Liss, Inc.

KEY WORDS

IUD, actinomyces, Pap smear

Human actinomycosis was first described by Israel.[1] The first report of genital actinomycosis infection was made in 1926 in a woman who was using an intrauterine pessary.[2] Henderson's[3] report in 1973 brought the association of pelvic actinomycosis and intrauterine contraceptive devices (IUDs) to the attention of gynecologists. Since then, actinomyces colonization has been described in otherwise healthy women without IUDs by Persson and Holmberg.[4]

Gupta and associates[5–7] first described the histomorphologic identification of the carriage of actinomyces-like organisms (ALOs) and their association with IUDs on Pap smears. Gupta[7] has demonstrated that Pap-stained cervicovaginal smear diagnosis of ALOs has both a high specificity (98%) and sensitivity (94%) when correlated with fluorescence antibody staining.

The management of IUD-associated ALOs identified on a cervicovaginal Pap smear, especially in an asymptomatic woman, is difficult, controversial, and without any uniform consensus. It ranges from leaving the IUD in situ to removal, with or without reinsertion of a new IUD or antibiotic treatment.[8–11]

Our study assesses some of the ways to manage ALOs diagnosed by cervicovaginal Pap smears in asymptomatic IUD users.

### MATERIALS AND METHODS

Data were compiled from records of patients attending the Temple University Hospital Family Planning Clinic from 1975 to 1985. We accumulated a list of 1,745 patients utilizing the IUD as a means of contraception. These patients' Pap smear results were reviewed for the presence of ALOs. One hundred seventy-three patients were found to have ALOs on their Pap smears. Pap smears were the only method used for diagnosis of ALOs. The patients' charts were then reviewed to determine the

Address correspondence/ reprint requests to Dr. Ashwin Chatwani, Temple University Hospital, Department of OB/GYN & RS, 3401 North Broad Street, 7-OPD, Philadelphia, PA 19140.

Received June 8, 1993
Accepted September 15, 1993

Clinical Study date of IUD insertion and removal and if antibiotics were taken while the IUD was in situ with presence of ALOs on their Pap smears. Criteria for inclusion in this study were: 1) Pap smear without ALOs prior to the insertion of the IUD; 2) Pap smear positive for ALOs after insertion of the IUD; 3) follow-up Pap smear within 6 months after treatment; and 4) asymptomatic status at the time of initial positive Pap smear.

The patients were divided into 4 groups according to their management:

*Group 1:* Removal of IUD alone
*Group 2:* Antibiotics alone
*Group 3:* Removal of IUD and antibiotics
*Group 4:* No treatment

It should be noted that removal of the IUD with or without antibiotics was often carried out for reasons other than treatment of cervical actinomyces: the presence of abnormal uterine bleeding (n = 27), abnormal uterine bleeding with concomitant unrelated infections (n = 27), a change to other forms of contraception (n = 31), or the desire for future fertility (n = 29). Patients in group 2 received antibiotics for concomitant infections and not ALOs.

Patients with negative follow-up Pap smears after treatment were classified as successes, and patients with persistent ALOs after treatment were deemed failures. A small number of patients in group 4 with ALOs were eventually lost to follow-up.

Antibiotics received by patients in groups 2 and 3 were divided into four groups: A) ampicillin, 500 mg 4 times a day for 7–10 days/penicillin, 250 mg 4 times a day for 7–10 days; B) doxycycline, 100 mg twice a day for 7 days/tetracycline, 500 mg 4 times a day for 7 days; C) metronidazole, 250 mg 3 times a day for 7 days; and D) multiple antibiotics (any combination of the aforementioned antibiotics).

Statistical analyses were performed using Epi Info (Centers for Disease Control, Atlanta, GA). The Mantel-Haenszel chi square formula was used. When a cell value of less than 5 was encountered, a 2-tailed $P$ value was obtained with the Fisher's exact test.

### RESULTS

There was a total of 173 patients with IUD-associated ALOs on cervicovaginal Pap smear. Table 1 details the results of the management in each of the four groups described.

**TABLE 1. Management of IUD-associated ALOs**

| Group | Type of treatment | No. of patients | Follow-up: Negative [no. (%)] | Follow-up: Positive [no. (%)] |
|---|---|---|---|---|
| 1 | Removal of IUD | 76 | 74 (97.4) | 2 (2.6) |
| 2 | Antibiotics | 38 | 14 (36.8) | 24 (63.2) |
| 3 | Antibiotics and removal of IUD | 38 | 38 (100) | 0 |
| 4 | No treatment | 21 | 3 (14.3) | 18 (85.7) |

Group 1 consisted of 76 patients who had ALOs on the Pap smear. Removal of the IUD alone gave a success rate of 97.4%. Two patients with persistent ALOs on their post-treatment smears were lost to follow-up. In group 2 all of the patients who failed initial antibiotic therapy had their IUDs removed subsequently and had a negative Pap smear within 2 weeks to 6 months of IUD removal. Eighteen patients in group 4 who continued to have positive smears were eventually lost to follow-up. All patients in this group had a minimum follow-up of 6 months.

The results of management with removal of IUD alone or removal of IUD with antibiotics were found to be significantly better than management with antibiotics alone ($P = 0$ and $P = 0$). There was no difference when removal of IUD alone was compared with IUD removal with antibiotics ($P = 0.31$).

There were 27 patients who had their IUDs removed because of abnormal uterine bleeding. Seventeen of these patients had IUD removal alone, while ten patients had IUD removal and antibiotics. All 27 patients had subsequent negative Pap smears.

The types of antibiotics used in group 2 patients are presented in Table 2. The ampicillin/penicillin group was not found to be different from the doxycycline/tetracycline and metronidazole groups ($P = 0.30$ and $P = 0.23$). However, patients who received multiple antibiotics had a better chance of cure when compared with patients who received doxycycline/tetracycline ($P = 0.04$).

### DISCUSSION

Our retrospective analysis indicates that the best form of treatment for IUD-associated ALOs is

**TABLE 2. Antibiotic therapy only**

| Group | No. of patients | Follow-up Negative [no. (%)] | Follow-up Positive [no. (%)] |
|---|---|---|---|
| Ampicillin/penicillin | 8 | 3 (37.5) | 5 (62.5) |
| Doxycycline/tetracycline | 12 | 2 (16.5) | 10 (83.3) |
| Metronidazole | 10 | 4 (40) | 6 (60) |
| Multiple antibiotics | 8 | 5 (62.5) | 3 (37.5) |
| Total | 38 | 14 (36.8) | 24 (63.2) |

removal of the IUD. Addition of antibiotics at the time of removal of the IUD does not increase the overall success rate. Seventy-four of 77 patients who had only removal of the IUD had subsequent negative Pap smears, and all 38 patients who had IUD removal and antibiotics had negative follow-up Pap smears. This difference was not statistically significant ($P = 0.31$).

Treatment with antibiotics without removal of the IUD gave an overall success rate of only 36.8%, which was significantly less than IUD removal or IUD removal and antibiotics ($P = 0$). As the presence of the IUD acts as the focus for this foreign-body-induced infection, its removal seems essential for successful therapy.

None of our patients with IUD-associated ALOs developed serious upper genital tract infection. This finding has also been reported by other authors.[4,8,9] However, women with IUD-associated ALOs on Pap smears of the cervix have been shown to have a 3.6-fold greater incidence of hospitalization for pelvic inflammatory disease (PID).[12] Tubo-ovarian abscesses have also been reported to be more common in women with IUDs and colonization of ALOs when compared with women with IUDs but not colonized with ALOs.[12] In our study, there were 27 patients who had their IUDs removed because of abnormal uterine bleeding. It is probable that this symptom was secondary to endometritis, but none of these patients had endometrial biopsy for confirmation.

Singh et al.[9] suggested expectant management in asymptomatic women if IUD-associated ALOs are noted on Pap smears. This recommendation is based on their findings of only 4.8% with positive cultures for ALOs in the endometrial biopsies, implying to them that there is only superficial colonization of this organism of the cervix and IUD. This policy of leaving the device in situ needs further evaluation. Mao and Guillebaud[8] have suggested removal and reinsertion of another IUD as satisfactory management of IUD-associated ALOs. In their study, seven patients who had IUDs removed for ALOs and a new IUD inserted at the same time had no evidence of ALOs on subsequent Pap smears. Spontaneous resolution of IUD-associated ALOs has also been reported.[8] This occurred in three of our patients.

Expectant management, as some investigators have advised, is often an attractive option. Even though spontaneous resolution does occasionally occur, it is recommended that patients with IUD-associated ALOs be managed with removal of the device. The addition of antibiotics does not significantly alter the success rate. These patients should be followed with repeat Pap smears any time after 2 weeks of treatment. Once a negative smear is obtained, consideration may be given to reinsertion of a new IUD.

### REFERENCES

1. Israel J: Neue Beobachtungen auf dem Gebiete der Mykosen des Menschen. Arch Pathol Anat 74:15, 1878.
2. Draper JW, Studdiford WE: Report of a case of actinomyces of the tubes and ovaries. Am J Obstet Gynecol 11:603–608, 1926.
3. Henderson SR: Pelvic actinomycosis associated with an intrauterine device. Obstet Gynecol 41:726–732, 1973.
4. Persson E, Holmberg K: A longitudinal study of actinomycosis Israelii in the female genital tract. Acta Obstet Gynecol Scand 63:207–217, 1984.
5. Gupta PK, Hollander DH, Frost JK: Actinomycetins in cervicovaginal smears: An association with IUD usage. Acta Cytol 20:295–297, 1976.
6. Gupta PK, Erozan YS, Frost JK: Actinomycetes and the IUD: An update. Acta Cytol 22:281–282, 1978.
7. Gupta PK: Intrauterine contraceptive devices: Vaginal cytology, pathologic changes and clinical implications. Acta Cytol 26:571–613, 1982.
8. Mao K, Guillebaud J: Influence of removal of intrauterine contraceptive devices on colonization of the cervix by actinomyces-like organisms. Contraception 30:535–544, 1984.
9. Singh MM, Ingham HR, Wadehra V, Morris K: Endometrial culture in IUD users with actinomycosis-like organisms (ALOs) on cervical smears. Br J Family Planning 15:3–6, 1989.
10. Hager WD, Majmudar B: Pelvic actinomycosis in women using intrauterine contraceptive devices. Am J Obstet Gynecol 133:60–63, 1979.

11. Bhagavan BS, Gupta PK: Genital actinomycosis and intrauterine devices: Cytopathologic diagnosis and clinical significance. Hum Pathol 9:567–578, 1978.

12. Burkman R, Schlesselman S, McCaffrey L, Gupta PK, Spence M: The relationship of genital tract actinomycoses and the development of pelvic inflammatory disease. Am J Obstet Gynecol 143:585–589, 1982.
